# Deceptive actions in combat sports: an antagonistic decision-making perspective from the lens of “*Shi*”

**DOI:** 10.3389/fpsyg.2026.1923989

**Published:** 2026-08-18

**Authors:** Zhang Wenfu

**Affiliations:** College of Physical Education, Hebei Normal University, Shijiazhuang, China

**Keywords:** combat sports, deceptive actions, decision-making, psychological momentum, SHI

## Abstract

Deceptive actions are prevalent in combat sports, yet existing research is limited by a focus on ball sports, fragmented single-level explanations, and a lack of cross-level integration. This paper introduces “*Shi*” (势)–a concept from Chinese martial arts denoting a dynamic strategic advantage emerging from posture, intention, and momentum–as an analytical lens to address these gaps. We propose an integrative framework in which “*Shi*” operates across biomechanical, cognitive, and dynamic levels. The framework’s core mechanism is a hypothesized cross-level cascade: deception is proposed to induce bodily imbalance, which is theorized to impair cognitive judgment, which in turn is predicted to undermine psychological momentum, forming a self-reinforcing downward spiral. This cascade mechanism distinguishes the “*Shi*” framework from existing single-level accounts and offers a novel perspective for understanding deception, anticipation, and decision-making in combat sports, generating empirically testable hypotheses for future research.

## 1 Introduction

In competitive sports, athletes frequently conceal information or provide misleading cues about their forthcoming action intentions. Concealing cues is referred to as disguised action, whereas actively providing false information is termed deceptive action (Güldenpenning et al., 2017). Raffan et al. (2024) recently extended this distinction by proposing a conceptual framework that separates deceptive movements into deceit (actively misleading an opponent) and disguise (concealing action intentions)–a distinction that informs the operationalization of Shi assessment in the present framework. Research on deceptive actions has been conducted across multiple sports, including cricket, rugby, football, soccer, and basketball (Güldenpenning et al., 2017). The core assumption underlying deceptive actions is that providing minimal or invalid information about one’s action intentions increases the likelihood of outperforming the opponent (Brault et al., 2012). Research on anticipation in sport has established that skilled athletes extract advanced information from opponents’ kinematics to guide their responses (Abernethy and Russell, 1987).

Existing research primarily revolves around three themes: (1) the role of expertise in the recognition of deceptive actions; (2) the cognitive mechanisms underlying the processing of deceptive actions; and (3) the ecological validity trade-offs between laboratory and field research (Güldenpenning et al., 2017). However, three core limitations characterize the existing literature. First, the empirical evidence on deceptive actions comes predominantly from ball sports such as basketball, rugby, and football (Güldenpenning et al., 2017), with combat sports receiving comparatively little empirical attention. In Güldenpenning et al.’s (2017) systematic review of 24 studies on deceptive actions in interactive sports, only two studies involved combat or martial arts contexts. Second, even in combat sports research, existing studies have largely focused on isolated aspects of deception–such as visual search strategies in karate (Williams and Elliott, 1999) or the kinematics of deceptive movements in boxing (Hristovski et al., 2006)–without integrating these findings into a comprehensive framework that links perceptual, cognitive, and dynamic processes. Third, no existing framework has yet explicitly integrated how bodily imbalance impairs cognitive judgment with how cognitive uncertainty amplifies the collapse of psychological momentum–that is, the cross-level cascade mechanisms that operate during continuous cycles of deception and counter-deception remain largely unexplored. The “*Shi*” (势) framework proposed below is designed to address these three limitations simultaneously by providing an integrative account of how biomechanical, cognitive, and dynamic levels couple and cascade in real-time combat interactions.

The concept of “Shi” (势) in Chinese martial arts provides a unique analytical lens for understanding this dynamic game. “Shi” is a core concept in traditional Chinese culture, spanning military strategy, politics, and the arts (Jullien, 1995), and is understood in martial arts traditions as a dynamic strategic advantage arising from the coordinated configuration of posture, momentum, and psychological state. Critically, “Shi” is characterized by three interrelated features: (a) holism: it is not reducible to any single dimension; (b) coupling: its three constituent levels mutually constrain and amplify each other; (c) dynamic game-theoretic evolution: it continuously shifts in response to the ongoing competitive interaction. These features distinguish “Shi” from static or single-level concepts and constitute the theoretical value of the framework proposed in this paper. Scholars have noted that “Shi” encompasses both objective and subjective dimensions, as well as both material and spiritual aspects (Zhang and Zhang, 2023). For the purposes of the present analysis, we operationalize “Substantive Shi” (shishi 势虚) and “Vacuous Shi” (shixu 势虚) as biomechanical states of postural balance and imbalance, respectively–the former referring to a state in which the body maintains a highly stable postural structure and force transmission system, and the latter to a state in which instability disrupts effective force transmission. This operationalization is a context-specific simplification for combat sports rather than an exhaustive account of the traditional philosophical concept, which also encompasses dimensions of form, energy, and intentionality (Zhang and Zhang, 2023). Our biomechanical focus is justified by the central role of postural control in combat effectiveness and by our aim to render the concept empirically tractable. At the psychological level, “Shi” concerns the perception, judgment, and utilization of the opponent’s intentions.

Before proceeding, it is important to clarify the relationship between the philosophical richness of “*Shi*” and its empirical operationalization in the present framework. As a traditional Chinese concept, “*Shi*” indeed carries holistic connotations that encompass physical form (*xing* 形), energetic readiness (*jin* 劲), and intentional/momentum states (*yi* 意 and *shi* 势). Our three-level analytical framework–biomechanical, cognitive, and dynamic–is designed to collectively capture these dimensions: the biomechanical level addresses the physical “form” and force transmission; the cognitive level (Theory of Mind/*Shi* assessment) addresses the “intentional” dimension; and the dynamic level (Psychological Momentum) addresses the “momentum/energy” dimension. Thus, no single level alone constitutes “*Shi*”–it is the integration and coupling of all three that defines the construct. The biomechanical operationalization of Substantive and Vacuous *Shi* is therefore best understood as a proxy indicator (rather than an exhaustive definition) of the physical foundation upon which the cognitive and dynamic dimensions operate. This proxy logic allows for empirical tractability without denying the ontological richness of the traditional concept–a distinction we revisit in the Limitations section.

This paper aims to propose a theoretical framework with “*Shi*” as the core analytical lens, re-examining the psychological mechanisms of deceptive behavior in combat sports, and synthesizing existing empirical findings into a novel theoretical framework through conceptual analysis.

## 2 The three analytical levels of “Shi”

We decompose the concept of “*Shi*” into three analytical levels, each of which interfaces with one or more psychological theories.

Before presenting the three levels, it is important to clarify that the analytical decomposition of “Shi” is not intended to replace existing concepts such as affordance, Theory of Mind, or psychological momentum. Instead, the value of the “Shi” framework lies in its capacity to integrate these dimensions–to show how bodily imbalance, cognitive misjudgment, and momentum collapse are not separate phenomena but mutually amplifying processes within a single competitive event. The three existing concepts each explain isolated aspects of deception, but none addresses how these aspects interact, cascade, and feedback upon each other in real time. Table 1 contrasts these existing concepts with the integrative logic of the “Shi” framework.

**TABLE 1:** Comparison of single-level theoretical concepts and the integrative *Shi* framework.

Concept	Level of analysis	What it explains	What it misses
Affordance (Gibson, 1979)	Biomechanical/perceptual	Action opportunities in the environment	How cognitive judgment and momentum interact with perceived opportunities
Theory of Mind (Van Overwalle and Baetens, 2009)	Cognitive	Inference of others’ intentions	How bodily instability impairs inference, how momentum biases inference
Psychological Momentum (Iso-Ahola and Mobily, 1980	Dynamic/motivational	Sequential performance advantage	How bodily imbalance and cognitive uncertainty generate or destroy momentum
Shi (present framework)	Integrative	How biomechanical, cognitive, and dynamic factors couple and cascade	Empirical validation across all three levels remains ongoing

### 2.1 Body–mechanics: “Shi” as balance and imbalance

Building on the operationalization introduced in the Introduction, we now examine the biomechanical level of “*Shi*” in greater detail. It bears reiterating that this biomechanical dimension is one of three interdependent levels; the cognitive and dynamic levels, discussed in subsequent sections, are equally essential to capturing the holistic nature of “*Shi*.” The biomechanical level alone is a proxy, not a substitute, for the full construct. At the biomechanical level, the core meaning of “*Shi*” pertains to postural balance. “Substantive *Shi*” corresponds to a state of high stability with a rational postural structure and force transmission system, whereas “Vacuous *Shi*” corresponds to a state of instability in which the postural structure is disrupted and force cannot be effectively transmitted. In combat sports, one of the core objectives of deceptive actions is to induce the opponent into a state of “Vacuous *Shi*.”

This analysis can be aligned with Gibson’s (1979) ecological psychology concept of “affordance,” which refers to the action possibilities that the environment offers to an individual. Within the ecological dynamics perspective on combat sports, combat athletes are driven by the perception of affordances for attack and defense. Two opponents self-organize into an “interpersonal synergy” in which the perceptions and actions of both athletes are coupled (Kimmel and Rogler, 2018; Krabben et al., 2019).

The “Maximum Grip” framework proposed by Ramsey et al. (2022) further advances this line of thinking. This framework posits that each athlete attempts to gain “grip” on the interactive process through the exploitation of information–an “epistemic grip” achieved by utilizing information across multiple timescales to grasp the interactive situation, thereby facilitating openness to relevant affordances. The Maximum Grip framework represents a significant advance in deception research because it recasts deception not merely as a perceptual-cognitive challenge but as a fundamentally interactional phenomenon: one athlete’s deception targets the other’s epistemic grip on the ongoing situation, disrupting their ability to perceive and act upon meaningful affordances (Ramsey et al., 2022). This interactional view is highly compatible with the Shi framework, which similarly posits that deception operates through the disruption of the opponent’s situated advantage. Where the Shi framework extends the Maximum Grip account is in specifying the mechanisms through which this disruption occurs–namely, the cross-level cascade from bodily imbalance to cognitive uncertainty to momentum collapse. In this sense, the Shi framework can be read as a mechanistic elaboration of the broader interactional principles articulated by Ramsey et al. (2022). Deceptive behavior constitutes a “misleading affordance” that invites the opponent to act upon manipulated information (Ramsey et al., 2022), consistent with Kimmel and Rogler’s (2018) assertion that affordances in competitive interactions can be misleading.

From the “Shi” perspective, the state of “Substantive Shi” means that the athlete possesses rich, genuine affordances for initiating effective attacks or defenses, whereas “Vacuous Shi” means that affordances are diminished. Postural imbalance not only restricts the possibilities for physical action but also is hypothesized to impair the accuracy of cognitive judgment–a prediction that draws indirect support from the dual-task literature, which demonstrates that postural control consumes attentional resources that would otherwise be available for higher-order cognitive processing (Woollacott and Shumway-Cook, 2002; Fraizer and Mitra, 2008). A deceptive action–such as a feigned punch intended to lure the opponent into an erroneous defensive reaction–constitutes a “misleading affordance” that leads the opponent to mistakenly believe that an opportunity for attack exists (Substantive Shi), thereby inducing them to enter a state of imbalance (Vacuous Shi). A successful deception is, in essence, a disruption of the opponent’s “Shi.”

### 2.2 Cognition–decision: “Shi” as intention inference and deception recognition

At the cognitive level, “*Shi*” concerns the perception, judgment, and utilization of the opponent’s intentions. The martial arts practice of “*Shi* assessment” (*shenshi* 审势), observing the opponent’s posture, movement, and expression to discern their psychological state and intentions–is a higher-order cognitive activity involving the rapid inference of others’ intentions.

This analysis can be aligned with Theory of Mind (ToM), which refers to the capacity to infer the mental states of others–beliefs, intentions, and desires (Van Overwalle and Baetens, 2009). In combat, athletes need to infer what the opponent is currently doing, what they are about to do, and whether they are pretending to do something. Action anticipation tasks activate the temporoparietal junction, medial prefrontal cortex, and precuneus, all of which are key nodes in the Theory of Mind network (Van Overwalle and Baetens, 2009). In research on deceptive actions, researchers distinguish between two types of deceptive movement strategies: (1) deception–coordinating an action to convey a false intention; and (2) disguise–coordinating an action to conceal veridical information that might provide critical cues for anticipation (Ramsey et al., 2022).

From the “*Shi*” perspective, “*Shi* assessment” is not merely about perceiving the opponent’s current postural state, but also about anticipating the trajectory of the opponent’s “*Shi*”–will the opponent’s next action lead toward “Substantive *Shi*” or “Vacuous *Shi*?” A deceptive action is effective precisely because it exploits the opponent’s psychological processes, leading them to falsely infer the deceiver’s true intentions and thus respond in ways that benefit the deceiver. Conversely, the capacity to resist deception (“*Shi* recognition,” *shishi* 识势) depends on more accurate Theory of Mind reasoning–discerning whether the cues provided by the opponent are genuine or misleading.

The “Shi assessment” process described above involves more than inferring the opponent’s intentions; it also entails judgments about risk and uncertainty. When a combatant evaluates whether an opponent’s movement is genuine or deceptive, they effectively assess the probability of different outcomes and the risks associated with each response option. This risk-evaluation component aligns with prospect theory (Kahneman and Tversky, 1979), which describes how individuals make decisions under uncertainty, and with research on expertise-based decision strategies in sport (Raab and Johnson, 2007).

### 2.3 Dynamics–game: “Shi” as the ebb and flow of psychological momentum

At the dynamic level, “*Shi*” is not static but rather a variable that continuously evolves over the course of an interaction. In Chinese martial arts theory, the gain or loss of competitive momentum is regarded as the decisive factor in determining victory. The essence of combat lies in the ongoing game of gaining and losing “*Shi*.” Deceptive actions are a highly valuable strategic tool precisely because they can alter the balance of “*Shi*” in an instant. A successful feint can disrupt the opponent’s rhythm, undermine their confidence, and shift the trajectory of the interaction.

This analysis can be aligned with Psychological Momentum theory (Iso-Ahola and Dotson, 2016; Briki et al., 2015). Psychological momentum is defined as an added or gained psychological power that changes interpersonal perceptions and influences an individual’s mental and physical performance (Iso-Ahola and Mobily, 1980). In a field study of racquetball tournaments, Iso-Ahola and Mobily (1980) found that winners of the first game had a 73.1% probability of winning the second game, demonstrating that psychological momentum confers a genuine competitive advantage. Accordingly, once psychological momentum is activated, it generates “*Shi*” that extends or develops in the direction of momentum.

From the “*Shi*” perspective, deceptive actions can be understood as a “momentum intervention” strategy. When the opponent is in a state of “Substantive *Shi*”–that is, possessing psychological momentum, sufficient confidence, and stable posture–a successful deception can: (1) disrupt their postural balance, rendering them in “Vacuous *Shi*”; (2) create uncertainty at the cognitive level, making them doubt their own judgment; and (3) interrupt their momentum at the psychological level, undermining their confidence. These three effects together achieve a transformation of “*Shi*,” shifting the advantage from the opponent to oneself.

Conversely, the capacity to resist deception also depends on a dynamic perception of “*Shi*.” An athlete capable of accurately “*Shi* recognition” not only perceives the opponent’s current “*Shi*” state but also anticipates its trajectory, enabling them to respond correctly before the deception unfolds.

### 2.4 Cross-level coupling: how the three levels interact

The three analytical levels presented above are not independent dimensions but are dynamically coupled in real time. Three pathways of cross-level interaction are particularly relevant for understanding deception in combat sports:

Pathway 1: Biomechanical → Cognitive. Postural instability (Vacuous *Shi*) is proposed to impair cognitive processing by increasing proprioceptive noise and consuming attentional resources that would otherwise be available for intention inference. This prediction is consistent with findings from the cognitive load and postural control literature, which have shown that the maintenance of postural stability competes for limited attentional resources (Woollacott and Shumway-Cook, 2002; Fraizer and Mitra, 2008). As we acknowledge, direct empirical evidence for this specific pathway in combat sports remains limited at present; the pathway thus constitutes a theoretically derived hypothesis that awaits future testing in representative combat settings. This means that a combatant who is physically off-balance is also more likely to misread the opponent’s intentions–a double disadvantage.

Pathway 2: Cognitive → Dynamic. Cognitive uncertainty–doubt about whether an opponent’s movement is genuine or deceptive–is hypothesized to undermine psychological momentum. When a combatant becomes uncertain about their judgments, they hesitate; hesitation breaks the rhythm of confidence that sustains momentum (Iso-Ahola and Mobily, 1980). Thus, deception does not only create a momentary cognitive conflict; it initiates a decline in momentum that persists across subsequent exchanges.

Pathway 3: Dynamic → Biomechanical. The loss of psychological momentum is predicted to further exacerbate bodily instability, particularly when athletes attempt to consciously monitor and correct their movements under pressure. Reduced confidence leads to diminished muscle tension and slower reaction times, which in turn compromise postural control. This is theorized to create a self-reinforcing downward spiral: bodily instability → cognitive impairment → momentum collapse → further bodily instability. This spiral is the mechanism through which a single successful deception can cascade into a decisive competitive advantage.

The cascading logic described above also operates in the opposite, positive direction. Just as deception is hypothesized to trigger a downward spiral, successful recognition of deception (“*Shi* Recognition”) is predicted to initiate an upward spiral: an athlete who correctly reads the opponent’s intention and maintains postural stability (Substantive *Shi*) reinforces cognitive confidence, which in turn bolsters psychological momentum; heightened momentum further stabilizes postural control and sharpens judgment, creating a self-reinforcing virtuous cycle. Thus, the “*Shi*” framework inherently captures the full dynamics of adversarial interaction: it explains both how deception degrades performance (negative cascade) and how skilled performance resists and recovers from deception (positive cascade). This bidirectional logic is central to understanding the continuous game of deception and counter-deception in combat sports.

Importantly, the strength and direction of these cross-level pathways are likely to be moderated by individual and contextual factors. First, expertise level plays a critical role: expert athletes may exhibit greater resistance to the cascade from bodily instability to cognitive impairment, as well-practiced postural control becomes more automated and less dependent on attentional resources (Beilock and Carr, 2001). For novices, however, the same degree of postural disruption may impose a heavier cognitive cost, amplifying the downward spiral. Second, the competitive context matters: the coupling effects may be stronger during high-stakes moments (e.g., tie scores or late in a match) when cognitive load is already elevated, and weaker during low-pressure phases when athletes have greater attentional reserves. Third, individual differences in psychological resilience and emotion regulation may moderate the impact of cognitive uncertainty on momentum: athletes with higher resilience may recover more quickly from judgment errors, whereas those with lower resilience may experience prolonged momentum collapse. These boundary conditions do not invalidate the cross-level coupling logic; rather, they specify the conditions under which the cascade is most pronounced. Future research should systematically examine these moderators to refine the *Shi* framework and enhance its predictive power.

Critically, these pathways are not merely additive but multiplicative: the combined effect of simultaneous disruption at multiple levels exceeds the sum of individual effects. This non-linear interaction is the hallmark of “*Shi*” as an emergent phenomenon–a competitive state that is more than the sum of its biomechanical, cognitive, and dynamic components.

Previous research has documented related cross-level effects. For example, research on psychological momentum has demonstrated that momentum states are sensitive to both performance outcomes and perceived competence (Iso-Ahola and Mobily, 1980; Iso-Ahola and Dotson, 2016). However, no existing study has directly tested the cross-level cascade proposed here–namely, that bodily imbalance → cognitive uncertainty → momentum decline → further bodily instability.

## 3 An integrative framework

Synthesizing the three levels above, we propose a “*Shi*”-driven integrative framework for antagonistic decision-making.

Each processing stage of the framework corresponds to a different psychological theory: the perception stage aligns with ecological affordance theory (Gibson, 1979); the evaluation stage aligns with Theory of Mind (Van Overwalle and Baetens, 2009); the decision stage aligns with risk decision theory (Kahneman and Tversky, 1979; Raab and Johnson, 2007); the action stage aligns with action control and response inhibition theory (Wickemeyer et al., 2025; Verbruggen and Logan, 2008); and the feedback stage aligns with psychological momentum theory (Iso-Ahola and Mobily, 1980). Together, these theories provide cross-level theoretical support for the “*Shi*” framework. The framework’s processing stages–perception, evaluation, decision, action, and feedback–form a cyclical loop:

Opponent’s Action (Information Input)

→ Perception Stage: Detecting kinematic cues–identifying whether the opponent is in Substantive *Shi* or Vacuous *Shi*, and detecting early signs of *Shi* transition

→ Evaluation Stage: Integrating biomechanical (postural), cognitive (intentional), and dynamic (momentum) cues to assess the current *Shi* configuration–one’s own vs. opponent’s *Shi* state. This is where cross-level coupling occurs: postural cues inform intention inference; intention uncertainty modulates momentum perception; momentum biases postural judgment.

→ Decision Stage: Judging the likely trajectory of *Shi* transformation–Will the opponent move from Substantive to Vacuous? Which action (attack, defend, or feint) best exploits the opponent’s Vacuous *Shi* while protecting one’s own Substantive *Shi*?

→ Action Stage: Executing an action designed to transform the *Shi* configuration–a genuine attack to consolidate one’s Substantive *Shi*; a feint to induce the opponent into Vacuous *Shi*; a defensive move to restore one’s own Substantive *Shi*

→ Feedback Stage: Opponent’s reaction reveals the new *Shi* configuration–Was the opponent induced into Vacuous *Shi*, or did they maintain Substantive *Shi*? The resulting *Shi* configuration becomes the new “information input” for the next cycle

This framework is not a unidirectional linear process but rather a real-time, cyclical interactive loop–each reaction from the opponent immediately becomes new “information input,” initiating a new cycle of “Shi” perception, evaluation, and decision-making.

The action stage described above is underpinned by action control and response inhibition processes (Verbruggen and Logan, 2008). In combat sports, where decision-making windows are extremely short, the ability to withhold a prepared defensive action or to initiate a deceptive offensive action depends on effective inhibitory control. Wickemeyer et al. (2025) demonstrated that successful inhibition of a defensive response to a basketball pump fake becomes increasingly difficult as the fake progresses toward its natural endpoint, suggesting that response inhibition operates under tight spatiotemporal constraints in sport contexts. This stage-specific theoretical grounding ensures that each phase of the *Shi* Decision Cycle is supported by established psychological mechanisms.

The Shi Decision Cycle (SDC) model differs from generic information-processing models in two ways. First, its core logic is not “perceive–decide–act” but rather “detect Shi configuration → assess transformation potential → act to transform Shi → perceive new configuration.” Second, the evaluation stage is inherently cross-level: postural, intentional, and momentum cues are not processed separately but integrated into a holistic judgment of the opponent’s Shi state.

The core contribution of this framework lies in its use of “*Shi*” as a cross-level mediating concept that connects the physical (biomechanical), mental (cognitive), and dynamic (game-theoretic) levels–precisely the integrative dimension that existing single-level concepts (e.g., focusing solely on affordance or solely on psychological momentum) have failed to fully address.

## 4 Theoretical implications

The proposal of the “*Shi*” framework not only integrates multiple theoretical perspectives but also advances existing research in three substantive ways.

### 4.1 Theoretical integration: extending existing frameworks with cross-level cascade

The “*Shi*” framework builds on and extends two existing ecological frameworks: the Maximum Grip framework (Ramsey et al., 2022) and the interpersonal synergy model (Krabben et al., 2019). The Maximum Grip framework posits that performers develop “epistemic grip” on the interaction by exploiting information across multiple timescales, and that deception operates by offering a “misleading affordance” that challenges the opponent’s grip (Ramsey et al., 2022). This framework significantly advances understanding of deception as an informational phenomenon. However, its focus remains primarily on how information is exploited, manipulated, and concealed; it does not address the biomechanical consequences of deception (i.e., how deception induces postural imbalance) or the dynamic consequences of deception (i.e., how deception disrupts psychological momentum). The interpersonal synergy model (Krabben et al., 2019), grounded in ecological dynamics, conceptualizes combat dyads as a single dynamical system in which two opponents self-organize into coupled perception-action units. This model provides a powerful framework for understanding how combatants co-adapt and synchronize their behavior during interaction. However, it focuses primarily on the emergent coordination between individuals at the dyadic level, without specifying how deception cascades across specific levels–from bodily imbalance to cognitive uncertainty to momentum collapse–or how these levels feedback upon each other. The “*Shi*” framework integrates insights from both approaches while extending them in novel directions. Consistent with Maximum Grip, it treats deception as a challenge to the opponent’s grip on the interaction; consistent with the interpersonal synergy model, it treats combat as a coupled dyadic system. The critical extension lies in specifying the cross-level cascade mechanism: deception-induced bodily imbalance (biomechanical) is proposed to impair cognitive judgment, which in turn is hypothesized to undermine psychological momentum, and the collapse of momentum is predicted to further exacerbate bodily instability, creating a self-reinforcing downward spiral. This cascade mechanism–which operates across biomechanical, cognitive, and dynamic levels–is absent from both the Maximum Grip and interpersonal synergy accounts and constitutes the primary theoretical contribution of the “*Shi*” framework.

### 4.2 Paradigmatic shift: from discrete stimuli to continuous adversarial dynamics

Previous research has predominantly examined deception in laboratory settings using single-action recognition tasks, reducing deception to “reaction time and accuracy in response to a single fake” (Güldenpenning et al., 2017). In this paradigm, deception is operationalized as a discrete event–a head fake, a pass fake, a feigned kick–and researchers measure changes in the opponent’s anticipatory performance before and after the event. While this approach has generated valuable empirical evidence regarding the cognitive mechanisms of deception, it captures only a snapshot of what makes deception effective in real combat. In actual adversarial interactions, the value of deception lies not in “fooling the opponent once” but in how it alters the trajectory of subsequent interactions. For example, Helm et al. (2020) found that when action information was obscured by disguise, individuals increasingly relied on situational probability information to guide their judgments–suggesting that deception operates in a dynamic informational environment rather than a static one. The “*Shi*” framework reframes deception as a game node within a continuous stream of interaction. A feint is effective not because it causes the opponent to make a single incorrect judgment, but because it simultaneously disrupts the opponent’s postural system (body), judgment system (cognition), and confidence rhythm (psychological momentum), thereby destabilizing the entire competitive configuration. This reorients deception research from the laboratory’s “single stimulus” paradigm back to the real, continuous context of antagonistic interaction, where deception and counter-deception unfold in rapid, adaptive cycles.

### 4.3 Practical redefinition: deception as levers for Shi transformation

The “*Shi*” framework also redefines deceptive actions from “momentary movements” to “levers for momentum shifts.” In traditional sport psychology research, deceptive actions have been operationalized as discrete events–a head fake, a pass fake, a feigned kick–with the primary outcome measure being whether the opponent was successfully deceived. This binary framing obscures the broader strategic function of deception. From the “*Shi*” perspective, the significance of a deceptive action lies not in the action itself but in its function as a lever for changing the course of interaction. A successful deception is valuable not because it “fooled” the opponent, but because it simultaneously achieves three objectives: (1) it pushes the opponent from “Substantive *Shi*” to “Vacuous *Shi*” at the bodily level; (2) it creates doubt about their own judgment at the cognitive level; and (3) it interrupts their momentum rhythm at the psychological level. It is the combination of these three effects that makes a deception a fulcrum for reversing the entire competitive situation–and this is precisely the meaning of “lever.” This reconceptualization has direct practical implications for coaches and athletes. Traditionally, training for deceptive actions has focused on the movements themselves–how to make fakes more convincing, how to conceal true intentions. However, Raffan et al. (2024) pointed out that existing training research has primarily emphasized perceptual training using video paradigms, lacking the interactivity of real antagonistic contexts, which limits the transfer of training effects. The “*Shi*” framework suggests that the goal of deception training should not simply be “executing an effective fake,” but rather actively creating opportunities for “*Shi*” transformation during confrontation.

To translate these principles into actionable training practices, we propose a two-phase training model that balances conceptual learning with ecological validity. This model is explicitly grounded in the principles of Representative Learning Design (RLD; Pinder et al., 2011), which emphasizes that training should preserve the informational and task constraints of real performance to facilitate skill transfer.

Phase 1: Offline Concept-Building (Explicit *Shi* Assessment Training). In early training stages, athletes can engage in video-based or post-sparring debriefing sessions in which they verbally report their assessment of *Shi* states–identifying moments when they perceived the opponent as Substantive or Vacuous *Shi*, and evaluating whether their own judgments were accurate. This phase serves a metacognitive scaffolding function: it makes the abstract concept of *Shi* explicit, establishes a shared vocabulary between coaches and athletes, and helps novices learn to attend to the relevant perceptual cues. We acknowledge that this phase is not representative of real competition, precisely because it suspends the tight perception-action coupling that defines live combat. However, its purpose is pedagogical rather than performance-simulative–it is a necessary preliminary step for conceptual acquisition.

Phase 2: Online Action-Based Assessment (Representative *Shi* Training). Once athletes have acquired the conceptual foundations, training should transition to representative designs that fully preserve perception-action coupling. In this phase, athletes are instructed to demonstrate their *Shi* assessment through action rather than verbal report. For example, upon detecting the opponent’s Vacuous *Shi*, the athlete must immediately execute a prescribed tactical response–such as forward pressure, angle attack, or postural reset–that exploits the perceived vulnerability. The accuracy of *Shi* assessment is thus inferred from the success or failure of the subsequent action, not from a verbal judgment. This design ensures that perception, decision, and action remain tightly coupled, consistent with RLD principles (Pinder et al., 2011). Coaches can manipulate task constraints (e.g., spatial area, time pressure, score differential) to progressively increase representative complexity.

In addition to this two-phase model, two complementary training approaches can be integrated into the representative phase. Timing-focused deception training involves athletes initiating feints at different phases of an opponent’s movement (early, mid, late) and observing the differential effects on the opponent’s defensive response, training sensitivity to timing as a critical parameter of deceptive effectiveness. Adaptive deception games require athletes to change their deceptive strategy in response to the opponent’s adjustments, promoting dynamic adjustment capacity. Both approaches are consistent with RLD principles when implemented in live, interactive contexts rather than isolated drill formats.

We emphasize that Phase 1 is a scaffolding step that should be phased out as athletes progress; the ultimate goal is to embed *Shi* assessment within the perception-action loop of live combat, where assessment and action are one and the same.

## 5 Implications for future research

The “*Shi*” framework proposed in this paper remains at the stage of theoretical construction, and its validity awaits empirical testing. We propose the following directions for future empirical investigation:

**Hypothesis 1** (Expert-Novice Differences in *Shi* assessment): Meyer et al. (2022) found that expert basketball defenders exhibit more efficient visual search patterns when facing shot fakes; Jackson et al. (2018) likewise found that expert football players differ significantly from novices in their judgment strategies when discriminating between genuine and deceptive actions. Extending these findings, we predict that in combat sports, experts will outperform novices specifically in integrating information across the three *Shi* dimensions–postural cues, intentional cues, and momentum cues–to form a holistic judgment of the opponent’s *Shi* state. The expert advantage should be particularly pronounced when information is incomplete or when postural and intentional cues are in conflict, as these conditions demand cross-level integration. Novices, by contrast, are expected to rely predominantly on single-domain cues (e.g., posture alone or movement kinematics alone).

**Hypothesis 2** (Effectiveness of Deception in Inducing Vacuous *Shi*): Well-designed feints that create “misleading affordances” will disrupt opponents’ postural balance (Vacuous Shi) more effectively than non-deceptive actions. Moreover, feints that simultaneously induce postural imbalance and create cognitive uncertainty (e.g., by conflicting with the opponent’s prior expectations) should produce greater Vacuous *Shi* effects than feints that target only one level. This hypothesis can be tested using motion capture technology–comparing the degree of center-of-pressure displacement and response times when opponents face deceptive versus non-deceptive actions, and contrasting single-level vs. multi-level deceptive strategies.

**Hypothesis 3** (Momentum Shift as a Function of Vacuous *Shi*): A successful deceptive action will be accompanied by significant changes in psychological momentum–the deceiver’s psychological momentum increases, while the deceived opponent’s decreases. The magnitude of this momentum shift is expected to correlate with the severity of Vacuous *Shi* induced: larger postural disruptions and greater cognitive uncertainty should predict greater momentum losses. This prediction can be tested by combining motion capture (for postural assessment), judgment accuracy measures (for cognitive assessment), and subjective psychological momentum scales (e.g., Briki et al., 2015).

**Hypothesis 4** (Cross-Sport Generalizability): The relative weight of the three *Shi* dimensions varies across combat sports. Specifically, the biomechanical dimension will explain more variance in grappling sports (e.g., wrestling, judo) where postural control is directly tied to match outcomes, whereas the cognitive and dynamic dimensions will play a larger role in striking sports (e.g., boxing, fencing) with faster action-reaction cycles.

**Hypothesis 5** (Cross-Level Coupling Effects): Based on the theoretical analysis of the cross-level cascade mechanism above, we predict that the coupling effects among the three levels of indicators will be stronger in deception contexts than in non-deception contexts. Specifically, this cross-level coupling hypothesis comprises three testable sub-predictions:

(a) Main Effect: Following a successful deceptive action, the deceived individual’s three levels of indicators should change synchronously–increased postural imbalance (biomechanical level), decreased accuracy of intention judgment (cognitive level), and decreased psychological momentum scores (dynamic level)–and the magnitudes of change across these three indicators should show significant positive correlations.

(b) Mediation Effect: Psychological momentum is expected to mediate the relationship between bodily imbalance and cognitive judgment accuracy. The impairment of cognitive judgment following postural disruption is at least partially driven by the collapse of psychological momentum, and this mediation effect should be significantly stronger in deception contexts than in non-deception contexts.

(c) Synergy Effect: Deceptive actions that simultaneously target the opponent’s postural balance and cognitive judgment will produce a greater impact on psychological momentum than deceptive actions targeting a single level–the combined effect should significantly exceed the sum of individual effects.

If the above main effect, mediation effect, and synergy effect are all empirically supported, this would indicate that “*Shi*” is an emergent holistic phenomenon in which its three levels change synchronously and couple with one another following deception. If the three levels vary independently, or if the mediation/synergy effects are not significant, the integrative “*Shi*” framework would be called into question.

Toward Operationalization: Measuring the *Shi* Construct

The theoretical framework proposed above generates clear predictions, but its empirical utility depends on the ability to operationalize and measure the three *Shi* dimensions. We offer the following preliminary measurement framework to guide future research:

Biomechanical dimension (Substantive/Vacuous *Shi*): Center of Pressure (CoP) displacement and Center of Mass (COM) velocity during combat stance and movement execution can serve as objective indicators of postural stability. Greater CoP displacement following a deceptive action would indicate successful induction of Vacuous *Shi*. Motion capture systems (e.g., Vicon) and force plates can provide high-resolution temporal data.

Cognitive dimension (*Shi* assessment accuracy): Time-locked judgment tasks–such as freezing video stimuli at the moment of deception onset and asking participants to judge the opponent’s action intention–can measure the accuracy of intention inference. Response time and confidence ratings can supplement accuracy as dependent variables (Jackson et al., 2018). The critical contrast is between deceptive and non-deceptive trials.

Dynamic dimension (*Shi* momentum): Psychological momentum can be assessed via adapted versions of the Perceived Momentum Scale (PMS; Briki et al., 2015) or via performance trajectory analysis–for example, examining the slope of point-by-point outcomes across a match. The combination of subjective (self-report) and objective (performance trajectory) measures would strengthen convergent validity.

Construct validation strategy: To establish that these three dimensions collectively form a unified “*Shi*” construct, future research should employ a Multitrait-Multimethod Matrix (MTMM) design or Confirmatory Factor Analysis (CFA) to test whether the three indicators load onto a single higher-order factor. Furthermore, the cross-level coupling predictions (Hypothesis 5) can be tested using cross-lagged panel models, which would examine whether biomechanical instability predicts subsequent cognitive judgment errors, which in turn predict subsequent momentum decline. If all three dimensions vary independently, the integrative *Shi* framework would be called into question.

## 6 Research limitations

Several limitations of the present framework should be acknowledged. First, the “*Shi*” framework is purely theoretical at this stage; all proposed mechanisms and hypotheses await empirical testing. Future research should operationalize the three *Shi* dimensions and test the predicted cross-level coupling effects using appropriate experimental paradigms.

Second, the concept of “*Shi*” originates from Chinese martial arts and is embedded in a specific cultural and philosophical tradition. Its construct validity and cross-cultural applicability in sport psychology research have yet to be established. The operationalization proposed here–focusing on biomechanical, cognitive, and dynamic dimensions–is a deliberate simplification that may not capture the full richness of the traditional concept, which also encompasses dimensions of form, energy, and intentionality. We have sought to mitigate this limitation by treating the three analytical levels as collective proxies for the holistic construct rather than exhaustive definitions. However, we acknowledge that this proxy logic represents a trade-off between philosophical fidelity and empirical tractability. Future research may develop richer operationalizations–for example, by incorporating physiological measures of energetic readiness (e.g., heart rate variability, muscle activation patterns) or phenomenological assessments of intentionality–to more fully approximate the traditional concept. For now, we emphasize that our framework is a scientific starting point, not a philosophical final word.

Third, the framework is primarily applicable to one-on-one combat sports (e.g., boxing, wrestling, MMA, judo), where close physical proximity and continuous interaction are defining features. Its applicability to team sports, racket sports, or other interactive sport contexts requires further investigation and may require contextual modifications. We discuss this boundary condition in greater detail in the following sections.

(a) Potential overlap with existing theories. As noted in the introduction, the individual dimensions of Shi–biomechanical, cognitive, and dynamic–overlap with existing constructs such as postural control, Theory of Mind, and psychological momentum, respectively. The Shi framework’s unique value does not lie in replacing these constructs but in hypothesizing that they are systematically coupled: that is, changes in one dimension predict changes in the others in a non-additive manner (Hypothesis 5). If future research finds only independent effects at each level–that is, if biomechanical instability does not predict cognitive judgment errors, or if judgment errors do not predict momentum decline–the Shi framework would be unnecessarily redundant. This is a genuine risk, and we offer it as a falsifiable prediction rather than a foregone conclusion.

(b) Cultural specificity and cross-cultural applicability. The term “*Shi*” originates from Chinese martial arts and is embedded in a specific cultural and philosophical tradition. While we have attempted to operationalize the construct using universal psychophysical mechanisms–postural control, intention inference, and momentum–the question remains whether the patterns of coupling hypothesized here generalize across cultures. It is possible that cultural differences in implicit coordination, competitive norms, or training practices affect the strength or direction of the cross-level cascade. Cross-cultural replication studies–for example, comparing combat athletes from Eastern and Western training traditions–would be essential to establish the framework’s generalizability.

(c) Generalizability across sport contexts. While the Shi framework was developed with one-on-one combat sports in mind, its core logic–a cross-level cascade from bodily imbalance to cognitive uncertainty to momentum collapse–may also apply to other interactive sports. In team sports (e.g., basketball, soccer), deceptive actions such as pass fakes or body feints could similarly disrupt an opponent’s postural and cognitive state. However, the presence of multiple interacting agents and spatial dynamics would require significant adaptation of the framework. In racket sports (e.g., tennis, badminton), the mediating role of the racket introduces distinct perceptual-motor constraints. We therefore offer the Shi framework as a starting point for cross-sport generalization, not as a finalized template. Adapting it to new contexts will require sport-specific validation.

(d) The psychological momentum literature cited here derives predominantly from non-combat sports. The extent to which momentum dynamics in combat sports differ in magnitude, time course, or underlying mechanisms remains an open question pending direct empirical investigation.

## 7 Conclusion

This paper operationalizes the Chinese martial arts concept of “*Shi*” into three interrelated levels–biomechanical (balance/imbalance), cognitive (intention inference), and dynamic (momentum shift)–and connects each level to existing psychological theories (ecological affordance theory, Theory of Mind, and psychological momentum theory), thereby constructing an integrative framework for understanding deceptive behavior in combat sports.

Building on existing accounts that have examined deception from informational (Ramsey et al., 2022) and dyadic (Krabben et al., 2019) perspectives, the core theoretical contribution of the “*Shi*” perspective lies in providing an integrative framework for understanding the dynamic game-theoretic essence of deceptive behavior in combat sports. Existing research has already established the effectiveness of deceptive actions, the role of expertise, and the associated cognitive mechanisms (Güldenpenning et al., 2017; Ramsey et al., 2022). The “*Shi*” framework further reveals how deception operates through “body–cognition–dynamics” cross-level cascade effects, and how opposing sides compete for strategic advantage through continuous cycles of deception and counter-deception during offensive and defensive transitions.

Beyond combat sports, the *Shi* framework also offers a culturally distinctive lens for studying antagonistic decision-making more broadly. It demonstrates that traditional Eastern strategic concepts can be operationalized and integrated with Western psychological theories, enriching the global theoretical toolkit for dynamic interaction research.
